# A Rare Case of Lumbar Hernia Secondary to an Iliopsoas Abscess: A Case Report

**DOI:** 10.7759/cureus.26967

**Published:** 2022-07-18

**Authors:** Yashjot Kaur, Mehul Sinha, Harsimrat Singh

**Affiliations:** 1 Department of Surgery, Government Medical College, Amritsar, Amritsar, IND; 2 Surgery, International Society for Chronic Illnesses, Vadodara, IND; 3 Medicine, International Society for Chronic Illnesses, Vadodara, IND; 4 Medicine, Kasturba Medical College, Mangalore, IND; 5 Department of Surgery, General Surgery, Government Medical College, Amritsar, Amritsar, IND

**Keywords:** aortic valve stenosis, hernia, surgical mesh, smokers, psoas abscess, lumbar hernia

## Abstract

Lumbar hernia is an uncommon condition that can either be congenital or acquired. Acquired lumbar hernia is further divided into primary, with no identifiable cause, and secondary, occurring due to previous trauma, infection, or surgery. Here, we present the case of inferior lumbar hernia in a 65-year-old Asian male who was a chronic alcoholic and smoker. He presented with a complaint of a longstanding swelling in the right lumbar region for five years and no other associated symptoms. The swelling was reducible, an expansile cough impulse was felt on palpation, and bowel sounds were heard on auscultation. A contrast-enhanced computed tomography scan revealed a 6.7 cm defect in the lateral abdominal wall in the right lumbar region with bowel loops, cecum, ascending colon, mesentery, and mesenteric artery seen herniating through the defect. There was a history of an iliopsoas abscess at the same site five years ago, which was treated with incision and drainage. The patient was advised for an open mesh repair but could not be operated upon due to coexisting aortic stenosis and regurgitation. Our impression, from this report, is that a chronic iliopsoas abscess tracking to the inferior lumbar region and the incision and drainage thereof, leading to a weakness in the abdominal wall, may be considered to be a cause of inferior lumbar hernia, with chronic smoking on part of the patient being a significant contributing factor for the abdominal muscle weakness. Therefore prompt and meticulous treatment of an iliopsoas abscess must be done to prevent this complication.

## Introduction

Lumbar hernia is a rare entity with approximately only 300 case reports published worldwide [1]. The lumbar region is divided into two triangles, namely, the superior lumbar triangle, also known as the Grynfeltt-Lesshaft triangle, and the inferior lumbar triangle, also known as Petit’s triangle [2]. The inferior lumbar triangle is bound inferiorly by the iliac crest, laterally by the external oblique muscle, and medially by the erector spinae muscle. The floor is formed by aponeurosis of the transversus abdominis muscle, internal oblique muscle, and lumbodorsal fascia, and the roof by superficial lumbodorsal fascia [2].

Lumbar hernias can be congenital (10-20%), arising from congenital muscle weakness, or acquired (80-90%), which can be spontaneous, also known as primary, or because of any preceding events such as trauma, infection, or postoperative, also known as secondary acquired hernia [2-4]. Lumbar hernias can be asymptomatic, presenting only as a flank mass or along with back or flank pain [5], or even severe pain if strangulation of the hernia has occurred. Lumbar hernia is treated surgically, and both open and laparoscopic approaches have been described each with its own advantages and disadvantages [4].

We report a case of unilateral right-sided inferior lumbar hernia secondary to an iliopsoas abscess encountered in a 65- year-old male who was a chronic alcoholic and smoker. The patient could not be operated upon because of the coexisting moderate aortic stenosis (AS) and aortic regurgitation (AR).

This case report was previously presented as an abstract at Revolutionizing Global Surgery, an annual Global Surg Network international conference on November 14, 2021.

## Case presentation

A 65-year-old Asian male presented to the surgery ward with a chief complaint of swelling in the right lumbar region for five years. The swelling was sudden in onset and had gradually increased in size over the years. The swelling was self-reducible, reducing on lying down in the left lateral position and reappearing on lying down supine or standing. There was no history of any previous trauma. There was no history of pain or fever associated with the swelling, which ruled out the possibility of strangulation. The patient was a chronic alcoholic and smoker for the past 30 years with two to three packs smoked per day.

The patient reported a history of an iliopsoas abscess at the same site five years ago, the etiology of which was not found. *Mycobacterium tuberculosis* was negative on investigations. There was a delay on part of the patient to seek medical care. The abscess was later treated with incision and drainage.

**Figure 1:**
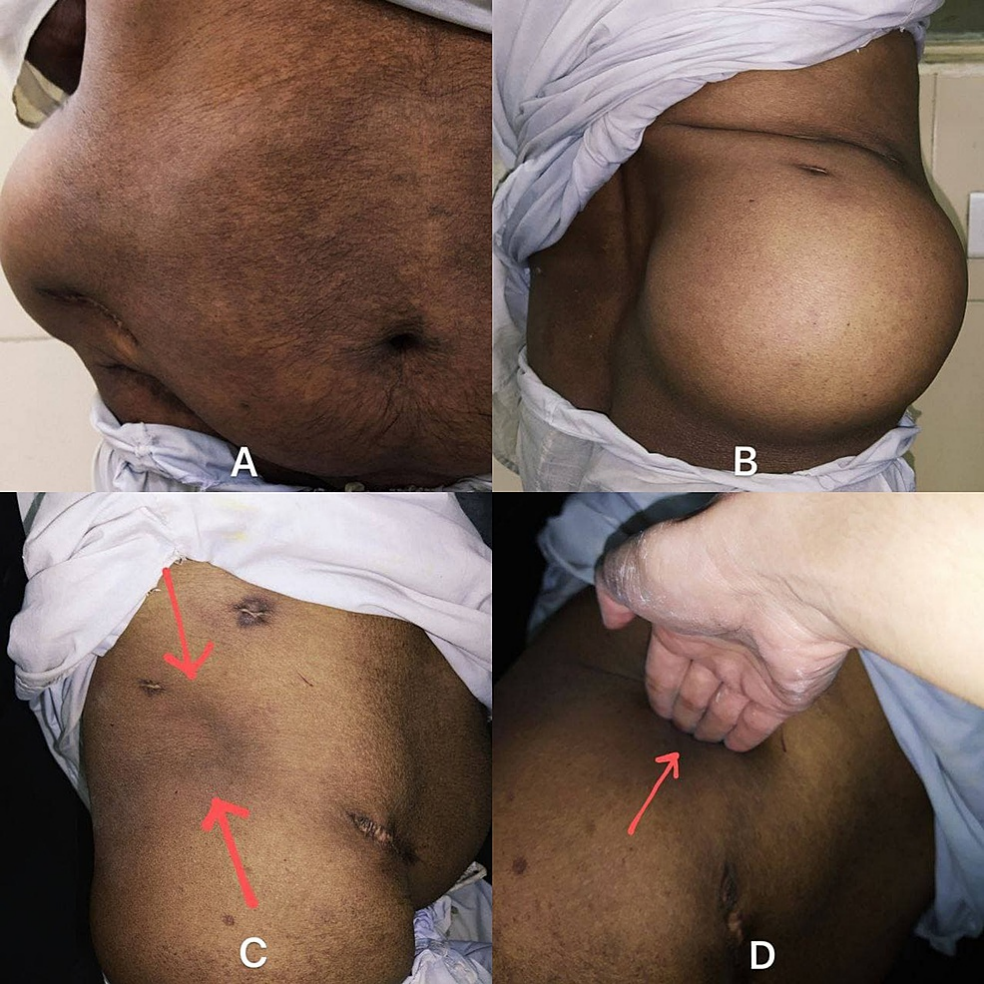
(A) Anterior view showing the right-sided lumbar hernia. (B) Lateral view showing the right-sided lumbar hernia. (C) Reducibility of the hernia with arrows pointing toward the defect. (D) Four fingers being insinuated into the defect.

Clinical examination revealed a swelling measuring 30 cm × 15 cm in the right lumbar region (Figures 1A, 1B). A scar mark suggestive of previous iliopsoas abscess drainage was present. There was no local rise in temperature or tenderness. Visible peristalsis was seen, and an expansile cough impulse was present. The swelling was doughy in consistency, with a smooth surface not attached to the skin, and normal overlying skin. On auscultation, bowel sounds were heard, suggestive of small bowel in the hernial sac. The swelling was reducible (Figure 1C). After reducing the swelling, a defect measuring approximately 5.5 cm × 1.5 cm was felt 1 cm above the iliac crest, and four fingers could be insinuated into the defect (Figure 1D).

Ultrasonography of the abdomen revealed a 5.5 cm × 1.5 cm defect in the right lateral abdominal wall, with mesentery and bowel loops seen herniating through it. A coincidental finding of a left-sided renal cyst 4.9 × 5.7 cm was also seen.

**Figure 2:**
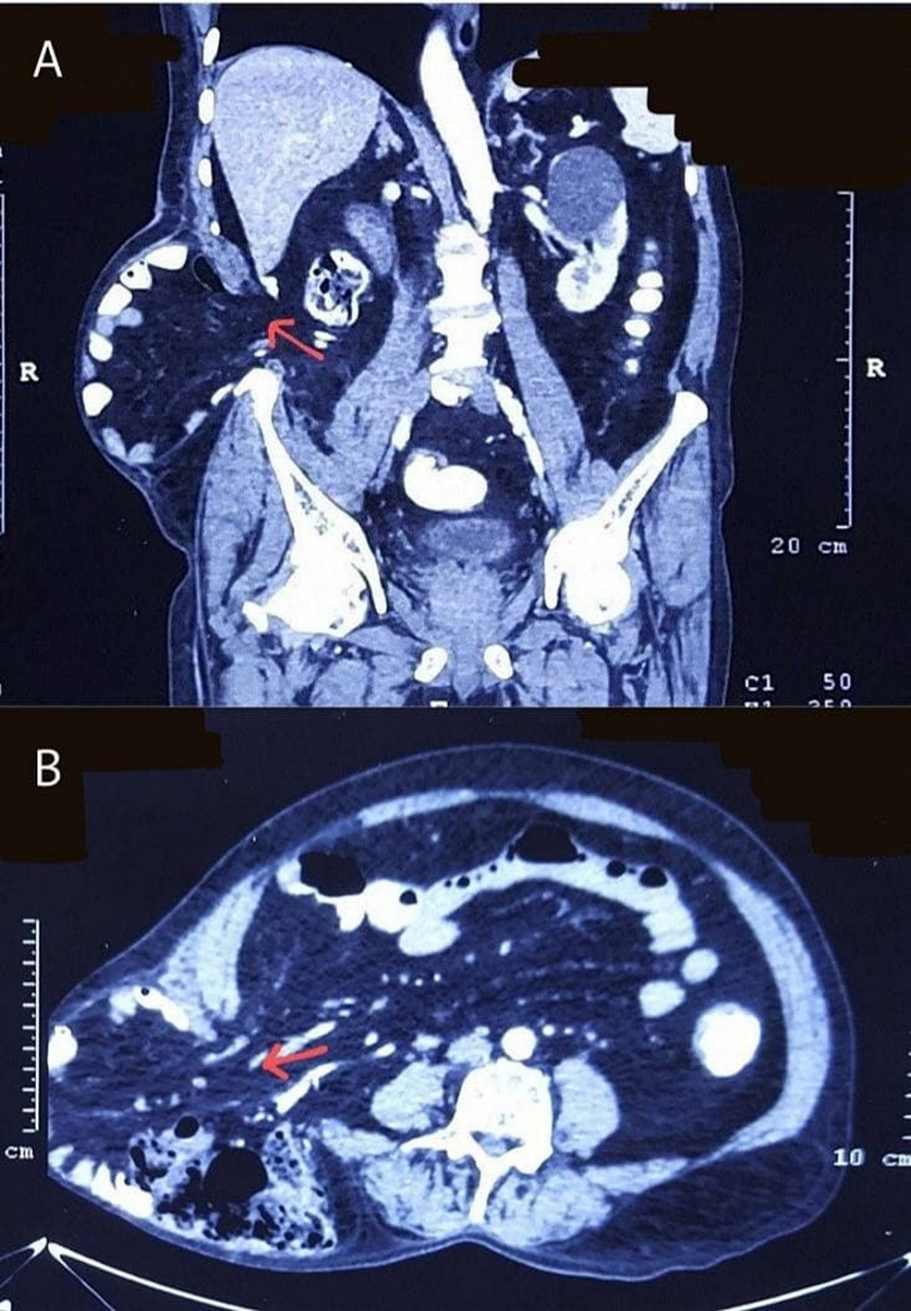
CECT abdomen with arrows pointing toward the defect and bowel loops seen herniating through the defect. (A) sagittal section. (B) Transverse section. CECT: contrast-enhanced computed tomography

Contrast-enhanced computed tomography (CECT) scan of the abdomen revealed a few small, hypodense, non-enhancing cystic lesions in segments IV and V of the liver, the largest measuring about 15 × 18 mm in size, suggestive of hepatic cysts. Few cortical cysts were seen in bilateral kidneys, the largest measuring 5.1 × 3.8 cm in the upper pole of the left kidney. As seen in Figures 2A, 2B, there was evidence of herniation of ileal loops, cecum, ascending colon, mesentery, and mesenteric vessels through a defect measuring 6.7 cm in the lateral abdominal wall in the lumbar region. The defect was seen between the quadratus lumborum and lateral abdominal wall muscles causing displacement of these muscles inferiorly, reaching up to the iliac crest.

Echocardiography showed grade one diastolic dysfunction with global hypokinesia, predominantly of the mid and distal septum, inferobasal left ventricular wall, and left ventricular apex. Mild septal hypertrophy and concentric left ventricular hypertrophy were present. The aortic root measured 2.4 cm. The aortic valve was thickened and restricted in motion. The maximum pressure gradient across the valve was 52 mmHg and showed trivial AG and moderate AS. The left ventricular ejection fraction was 55%.

Depending on the clinical and radiological findings, a diagnosis of right-sided lumbar hernia was made. Based on the findings, the patient was advised for an open mesh repair. He was referred to a cardiologist for the coexisting AG and AS and was recommended aortic valve replacement before proceeding with the open mesh hernia repair; however, the patient elected to avoid the surgery due to financial constraints. The patient is being followed up fortnightly, and there are no fresh complaints of skin changes or pain at the hernia site.

## Discussion

Lumbar hernia is an uncommonly encountered condition that can be divided into two categories, namely, superior and inferior lumbar hernia. It usually presents as a swelling in the lumbar region. While it is mainly asymptomatic, it may present with pain, backache, or strangulation, presenting as an acute abdominal emergency [2,5]. Our case of inferior lumbar hernia presented only with a swelling in the right lumbar region and no other associated symptoms, with pre-diagnosed AR and AS. The only positive history was a right-sided iliopsoas abscess, for which the patient sought treatment late in the course of the disease which may have led to the development of weakness of the abdominal muscles. Furthermore, the incision and drainage might have added to the weakness. In addition, the fact that the patient was a chronic heavy smoker might have predisposed him to abdominal muscle weakness. Hence, the most probable etiology for the development of the hernia was secondary to the abscess and the incision and drainage thereof, with chronic smoking on part of the patient being a significant contributing factor to the abdominal muscle weakness.

Lumbar hernias can easily be diagnosed based on clinical and radiological findings, with a CT scan being the gold standard [6]. While making a diagnosis, differentials such as retroperitoneal mass, sarcoma, lipoma, and a cold abscess must be ruled out [3,6-8]. Ultrasonography and magnetic resonance imaging can also help in confirming the diagnosis. In our case, the expansile cough impulse, reducibility of the swelling, the CT scan demonstration of the defect, and bowel loops in the sac confirmed the diagnosis of the hernia and ruled out other differentials.

The preferred treatment of a lumbar hernia is an open prosthetic mesh repair, but laparoscopic mesh repair is being increasingly considered [8]. The only pitfall is that parietal peritoneal closure cannot be done using a laparoscopic approach [1,6,9,10]. Primary repair of the defect is not preferred as it has tension [11], with high chances of recurrence. In our case, because the patient had AG and AS, we preferred an open approach to a laparoscopic one as carbon dioxide insufflation may decrease the venous return further aggravating the pre-existing heart condition. We planned to perform an open synthetic mesh repair, with the mesh placed extraperitoneal, aiming for a tension-free repair. Because the hernia was large, we planned to anchor the mesh inferiorly to the iliac crest, superiorly to the ribs, and dorsally, if possible, to the psoas muscle. The difficulty we expected while operating on the case is the loss of domain which could cause serious postoperative complications and repair site recurrence owing to the large size of the hernia. Unfortunately, the surgery could not be performed because the patient was not deemed fit for anesthesia.

## Conclusions

Lumbar hernias are extremely rare and present as a swelling or mass in the lumbar region. They can be diagnosed based on clinical and radiological findings, with a CT scan being the gold standard. A lumbar hernia occurs most commonly due to secondary causes such as trauma, infection, or surgery. Through this report, we conclude that a chronic iliopsoas abscess tracking to the inferior lumbar region and the incision and drainage thereof, leading to a weakness in the abdominal wall, may be considered as a cause of the inferior lumbar hernia, with chronic smoking on part of the patient being a significant contributing factor for the abdominal muscle weakness. Treatment varies depending on the patient variables and needs to be individualized. Mesh repair of the hernia is the most commonly preferred method which can be done either laparoscopically or by open repair. Primary repair of the defect is usually not preferred in a large hernia because of the high risk of recurrence. We report this case because a lumbar hernia secondary to an iliopsoas abscess has not yet been documented in the literature and more studies investigating this complication are required.
